# Steerable sheath visualizable under 3D electroanatomical mapping facilitates paroxysmal atrial fibrillation ablation with minimal fluoroscopy

**DOI:** 10.1007/s10840-022-01332-8

**Published:** 2022-08-10

**Authors:** Anil Rajendra, Tina D. Hunter, Gustavo X. Morales, Paul Zei, Lee Ming Boo, Allyson Varley, Jose Osorio

**Affiliations:** 1 Alabama Cardiovascular Group, Suite 720, Birmingham, AL 3686 USA; 2 CTI Clinical Trial Services, Covington, KY USA; 3 Brigham and Women’s Hospital, Boston, MA USA; 4 Biosense Webster, Inc, Irvine, CA USA; 5 Heart Rhythm Clinical and Research Solutions, Birmingham, AL USA

**Keywords:** Vizigo, Visualizable sheath, Atrial fibrillation, Catheter ablation, Low fluoroscopy

## Abstract

**Background:**

Advances in technology and workflows have facilitated substantial reductions in fluoroscopy utilization and procedure times for atrial fibrillation (AF) ablations. A recently available steerable sheath, visualizable on a 3D electroanatomical map (EAM), may further simplify low/zero fluoroscopy ablation workflows by facilitating understanding of the relative positions of the catheter and sheath. The objective of this study was to demonstrate feasibility, safety, procedural efficiency, and clinical effectiveness of incorporating the new visualizable sheath into a low-fluoroscopy workflow.

**Methods:**

Consecutive *de novo* paroxysmal AF procedures were performed with a porous tip contact force catheter at a high-volume site between January 2018 and May 2019. Procedures performed with and without the VIZIGO™ EAM-visualizable sheath (Vizigo) were compared. All ablations employed the same standardized low-fluoroscopy workflow. Statistical analyses employed stabilized inverse probability of treatment weights (IPTW) to balance cohorts by operator and key patient characteristics.

**Results:**

Cohorts of 142 Vizigo and 173 non-Vizigo patients were similar at baseline. Use of the Vizigo sheath was associated with approximately 10% improvement in catheter stability (*p* = 0.0005), 16% reduction in radiofrequency time (*p* < 0.0001), and 7% fewer ablations that used fluoroscopy (*p* = 0.0030). There was one cardiac tamponade in each cohort and no deaths, atrioesophageal fistulas, or strokes. Single-procedure freedom from atrial arrhythmia recurrence through 12 months was similar between cohorts (*p* = 0.9556).

**Conclusions:**

Use of a 3D EAM-visualizable sheath resulted in improved catheter stability, reduced radiofrequency time, and more procedures performed without fluoroscopy, without compromise to safety or effectiveness.

**Supplementary Information:**

The online version contains supplementary material available at 10.1007/s10840-022-01332-8.

## Introduction

Atrial fibrillation (AF) catheter ablation has become increasingly efficient due to advances in technology and workflows. Traditionally, catheter ablation procedures relied on the use of fluoroscopy to monitor catheter access and transseptal puncture and confirm catheter placement/location [1]. Steerable sheaths are often used to facilitate catheter positioning, stability, and tissue contact in the left atrium (LA) during ablation procedures and have been shown to increase clinical success [2, 3]. However, fluoroscopy is often required to confirm sheath location and its relation with the ablation catheter, resulting in radiation that presents health risks for both the patient and operator [4–9].

**Fig. 1:**
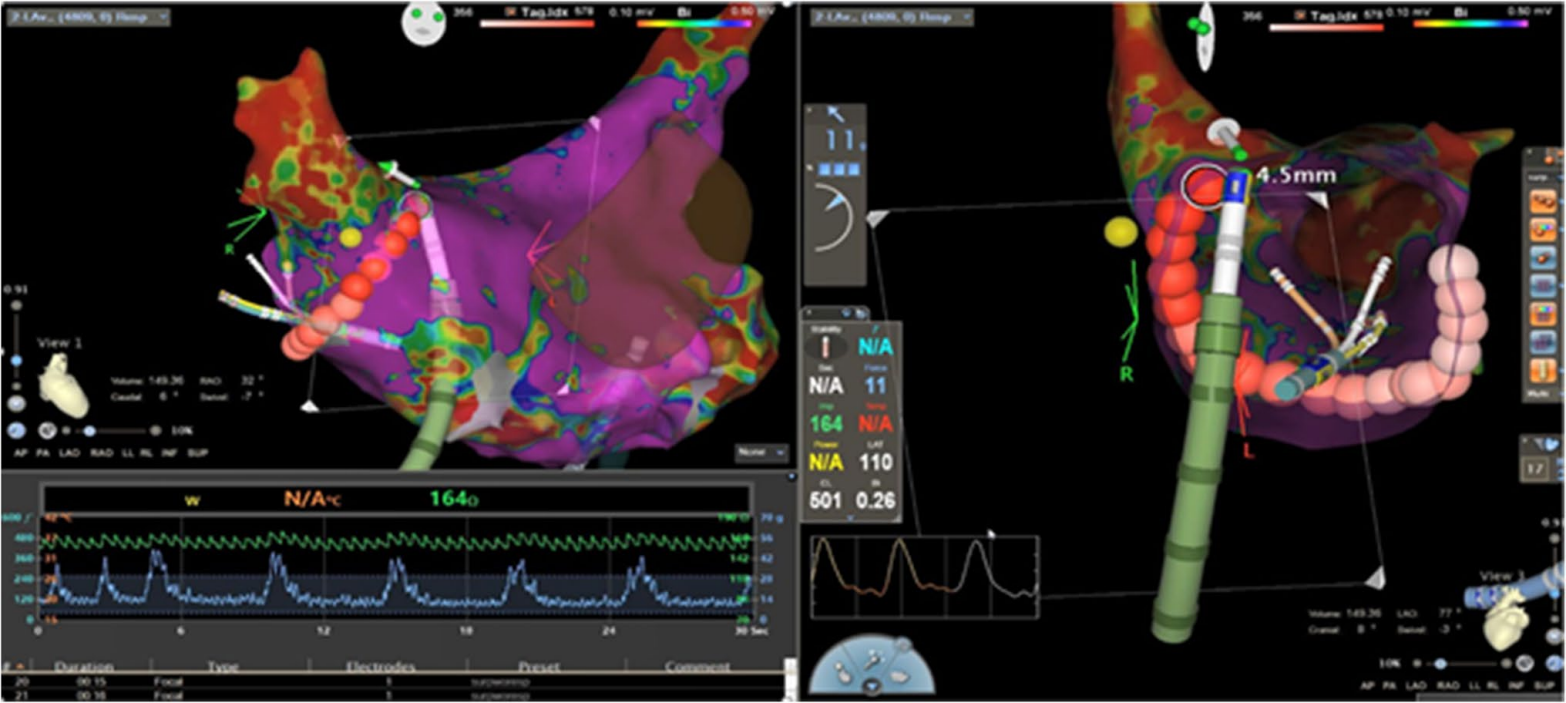
Real-time visualization of Vizigo on EAM during paroxysmal atrial fibrillation

Technological advances, specifically intracardiac ultrasound and 3D electroanatomic mapping (EAM) systems, have enabled catheter ablation procedure workflows to be transformed such that fluoroscopy utilization is reduced or eliminated without compromising safety or efficacy outcomes [1, 10, 11]. The new CARTO VIZIGO® bi-directional guiding sheath (Vizigo; Biosense Webster, Inc., Irvine, CA) is a novel steerable sheath that can be visualized with a 3D EAM system (Fig. 1). Direct visualization on EAM systems, paired with the smooth tip-to-dilator transition, facilitates entry into the LA during transseptal access without the need for supplemental fluoroscopy [12].

The current study documents the initial real-world experience of a single high-volume electrophysiology practice using this new sheath in conjunction with the CARTO® 3 System (CARTO; Biosense Webster, Inc., Irvine, CA) for radiofrequency (RF) catheter ablation procedures in a paroxysmal AF (PAF) population. The objective of the study was to demonstrate the feasibility, safety, procedural efficiency, and clinical effectiveness of incorporating this new visualizable sheath into a low-fluoroscopy workflow.

## Methods

### Study design

This was a non-randomized cohort study based on a retrospective analysis of patient-level data that was prospectively collected for ablation patients enrolled in the REAL AF Registry [13]. The registry enrolls consecutive adults (≥ 18 years) ablated with a contact force (CF) catheter at a participating site, excluding any patients enrolled in a clinical trial that would prescribe any aspect of their treatment.

The study population included patients having *de novo* PAF ablation performed by one of three operators at a single high-volume site between January 2018 and May 2019. Cohorts of interest were defined by procedures that utilized a Vizigo sheath (Vizigo cohort) versus those that did not (non-Vizigo cohort). All patients underwent their first left atrial ablation with the THERMOCOOL SMARTTOUCH® SF Catheter (STSF; Biosense Webster, Inc., Irvine, CA) and were evaluated according to standard clinical practices at the study site. Approval was obtained from the WCG Institutional Review Board.

### Ablation procedures

All patients were sedated using a previously described anesthesia protocol [14].

A decapolar deflectable catheter was placed in the coronary sinus. Intravenous heparin was administered before and after transseptal catheterization to target an activated clotting time of > 350 s. Transseptal catheterization was performed with a Fast-Cath SL2 preformed sheath (SL2; St. Jude Medical, Inc., St. Paul, MN) and a Brockenbrough needle and guided by intracardiac echocardiography (ICE) without fluoroscopy, as previously described [15]. When transseptal access was achieved, a J-tipped wire was advanced to the left superior pulmonary vein (PV), guided by ICE, and the trajectory of the wire across the fossa ovalis was marked with the Cartosound Module (Biosense Webster, Inc., Irvine, CA).

The ablation catheter was then advanced to the LA through the single transseptal puncture, following the wire trajectory, guided by ICE and Cartosound. In the Vizigo cohort, the steerable sheath was advanced into the left atrium over the ablation catheter. In contrast, a short Pinnacle introducer sheath (Terumo Medical Corporation, Elkton, MD) was used in the non-Vizigo cohort without a long sheath. The phrenic nerve was identified from within the right superior PV prior to the use of long-acting paralytics. At this point, the SL2 sheath was readvanced into the LA and a multipolar Pentaray Catheter (Biosense Webster, Inc., Irvine, CA) was introduced for mapping under the guidance of the EAM system. The LA geometry and voltage were acquired with CARTO, using parameters designed for rapid acquisition of geometric and voltage data. The ventilator inspiratory to expiratory ratio was changed to 1:4 in order to prolong the expiratory phase during mapping and atrial pacing was performed at 500 ms [14]. The multipolar catheter was positioned in the right upper vein after LA mapping was concluded, while the SL2 sheath was brought back into the inferior vena cava. Next, the ablation catheter sheath was reintroduced into the LA while the ablation catheter was fixed in the middle of the LA.

RF ablations were performed with the STSF catheter and PVs were isolated by wide area circumferential ablation (WACA), using the CARTO VISITAG™ Module with maximum location stability range of 2.5 mm and minimum stability time of 4 s. Visitag Surpoint (i.e., Ablation Index) was adopted in September 2018, with targets of 350 at the posterior wall and 500 at the anterior wall. CF and impedance were monitored in real time and CF was held between 10 and 20 g during ablation. Ablation was performed using a point-by-point technique, with the catheter moved to the next desired location during the last 1–2 s of each ablation lesion. After PVI was confirmed, a single 18 mg bolus of adenosine was injected intravenously to assess dormant conduction while sequentially mapping each PV antra. If dormant conduction was detected, further ablation was performed until the PVs were re-isolated. Thereafter, continuous isoproterenol infusion was administered at up to 20 mcg/min to identify any PV reconnection or non-PV triggers for 20 min after the last ablation lesion. If non-PV triggers were identified, they were targeted for additional RF ablation.

The CARTOUNIVU Module was used to integrate a fluoroscopy image with the EAM system as the background for understanding the spatial relation of implantable cardiac devices. Operators scrubbed in after the baseline images were obtained in these cases. This workflow did not require operators to wear a lead apron.

### Catheter stability assessment

In addition to the registry data, case data from CARTO was downloaded for the included ablation procedures from the CARTONET™ cloud-based storage and artificial intelligence analytics platform to evaluate catheter stability. Supplemental CARTO case data was available for 285 of the ablations (90.5%), of which 256 had complete location stability values. The stability values used for analysis represent the mean catheter displacement during the first 90% of the ablation time for each lesion. This allows for elimination of the purposeful catheter motion that occurs in the last 1–2 s as the catheter is repositioned to the next lesion location.

### Follow-up

Patients were followed for a year post-procedure to capture serious procedure-related complications, atrial arrhythmia recurrence, and reablation. Patient follow-up visits were scheduled at 10–12 weeks, 6 months, and 12 months, with all data collected using standardized forms. Cardiac event monitoring (e.g., Zio patch) was performed for a 96-h period at 6 and 12 months and as needed for symptoms, except for patients who already had an implantable cardiac device that could be queried to capture arrhythmias.

### Study outcomes

Measures of safety, procedural efficiency, and clinical effectiveness were compared between the Vizigo and non-Vizigo cohorts. The primary clinical effectiveness outcome was defined as single-procedure freedom from any post-blanking (90 days) atrial arrhythmia recurrence lasting longer than 30 s through the 12-month visit. Acute effectiveness was defined as achieving PVI, which was verified by adenosine and isoproterenol challenge. Procedure-related complications, fluoroscopy utilization, procedure times (including PV RF time, total RF time, and total procedure time), and reablations at any time were also reported.

### Statistical methods

The new Vizigo sheath was not adopted concurrently by operators of varying experience levels at the study site, leading to cohorts that were imbalanced with respect to a known confounder of both procedural efficiency and clinical effectiveness outcomes. In order to create the balance required for statistical inference, stabilized inverse propensity of treatment weights (IPTW) were utilized in the analysis of study outcomes. Propensity scores were calculated from a logistic regression model, representing the probability of a patient being ablated with Vizigo given the operator performing the ablation, while also adjusting for baseline age, sex, and CHA_2_DS_2_-VASc score. The stabilized IPTWs were then calculated from the propensity scores. IPTW weighting is used to adjust for imbalances in confounding variables across cohorts and produce unbiased estimates of average treatment effects, whereas the stabilized version also preserves the original sample size, leading to appropriate type I error rates and variance estimates [16–18]. Stabilized IPTW weighting was applied to all statistical models and statistical comparisons of outcomes across cohorts in order to prevent the effect of operator experience from confounding the effect of interest, namely, Vizigo vs. non-Vizigo cohort.

Weighted procedural outcomes were summarized with counts and percentages for categorical data and with means and standard deviations for continuous data. Statistical comparisons of these outcomes across cohorts used Pearson chi-square tests for categorical variables and *t*-tests for continuous variables. The Satterthwaite approximation was used for *t*-tests when variances were unequal across cohorts at a significance level of 0.05.

Clinical success was defined as single-procedure freedom from atrial arrhythmia recurrence after a 90-day blanking period. Several factors were explored as potential predictors of clinical success, including patient characteristics, procedural details, early recurrence within the blanking period, and Vizigo utilization. Patient characteristics of interest included sex, age, comorbid conditions, cardiac measures, and risk scores. Procedural details of interest included the lesion set utilized, substrate modifications, first pass isolation of PVs, and acute PV reconnection.

Categorical predictors of clinical success were explored individually with weighted Kaplan-Meier models and the relative risks were calculated for each predictor from weighted counts. Continuous predictors were explored via single-variable weighted Cox regression models. The primary predictor of interest was the sheath cohort (Vizigo vs. non-Vizigo ablations), but baseline patient characteristics, procedural details, and recurrences within the blanking period were also tested for association with success.

## Results

### Baseline patient characteristics and ablation detail

A total of 315 adult PAF patients ablated between January 2, 2018, and May 30, 2019, met all inclusion and exclusion criteria (Vizigo cohort: 142; non-Vizigo cohort: 173). Baseline patient characteristics were largely similar across the cohorts (Table 1). All patients received PVI and lesion sets included additional ablation in 66.5% of patients, most of which included cavotriscuspid isthmus. PVI-only ablation strategy was more prevalent in the Vizigo cohort (Table 1).

**Table 1:** Baseline patient characteristics and procedural details (unweighted)

Baseline characteristic	Vizigo (*N* = 142)	Non-Vizigo (*N* = 173)	*P*-value
Gender, male	65 (45.8%)	97 (56.1%)	0.0689
Age (years)	63.4 ± 11.9	63.9 ± 12.7	0.7146
Baseline antiarrhythmic drugs	81 (57.0%)	105 (60.7%)	0.4785
Previously failed antiarrhythmic drugs	54 (38.0%)	50 (28.9%)	0.0747
Pre-ablation oral anticoagulation	116 (81.7%)	149 (86.1%)	0.2836
Left ventricular ejection fraction (%)	58.7 ± 6.6	57.0 ± 6.0	0.0375
Left atrial diameter (cm)	3.9 ± 0.6	3.9 ± 0.6	0.3942
Congestive heart failure	6 (4.2%)	12 (6.9%)	0.3023
Hypertension	104 (73.2%)	112 (64.7%)	0.1059
Diabetes	16 (11.3%)	34 (19.7%)	0.0427
Prior stroke or transient ischemic attack	15 (10.6%)	24 (13.9%)	0.3749
Vascular disease	20 (14.1%)	41 (23.7%)	0.0317
CHA2DS2-VASc score	2.5 ± 1.5	2.6 ± 1.6	0.4424
HAS-BLED score	1.4 ± 0.8	1.4 ± 0.9	0.7327
Ablation procedure detail			
Lesion set			< 0.0001
PVI only	74 (52.1%)	30 (17.3%)	
PVI + CTI	53 (37.3%)	111 (64.2%)	
PVI + CTI + substrate modification	11 (7.7%)	25 (14.5%)	
PVI + substrate modification	3 (2.1%)	3 (1.7%)	
Missing	1 (0.7%)	4 (2.3%)	
Substrate modification at posterior wall	7 (4.9%)	23 (13.3%)	0.0118
Substrate modification at mitral isthmus	2 (1.4%)	8 (4.6%)	0.1053

Values are reported as mean ± standard deviation for continuous variables and percentages for categorical variables. *P*-values are from chi-square tests for categorical variables and *t*-tests for continuous variable. *PVI*, pulmonary vein isolation; *CTI*, cavotricuspid isthmus

### Procedural outcomes

**Fig. 2:**
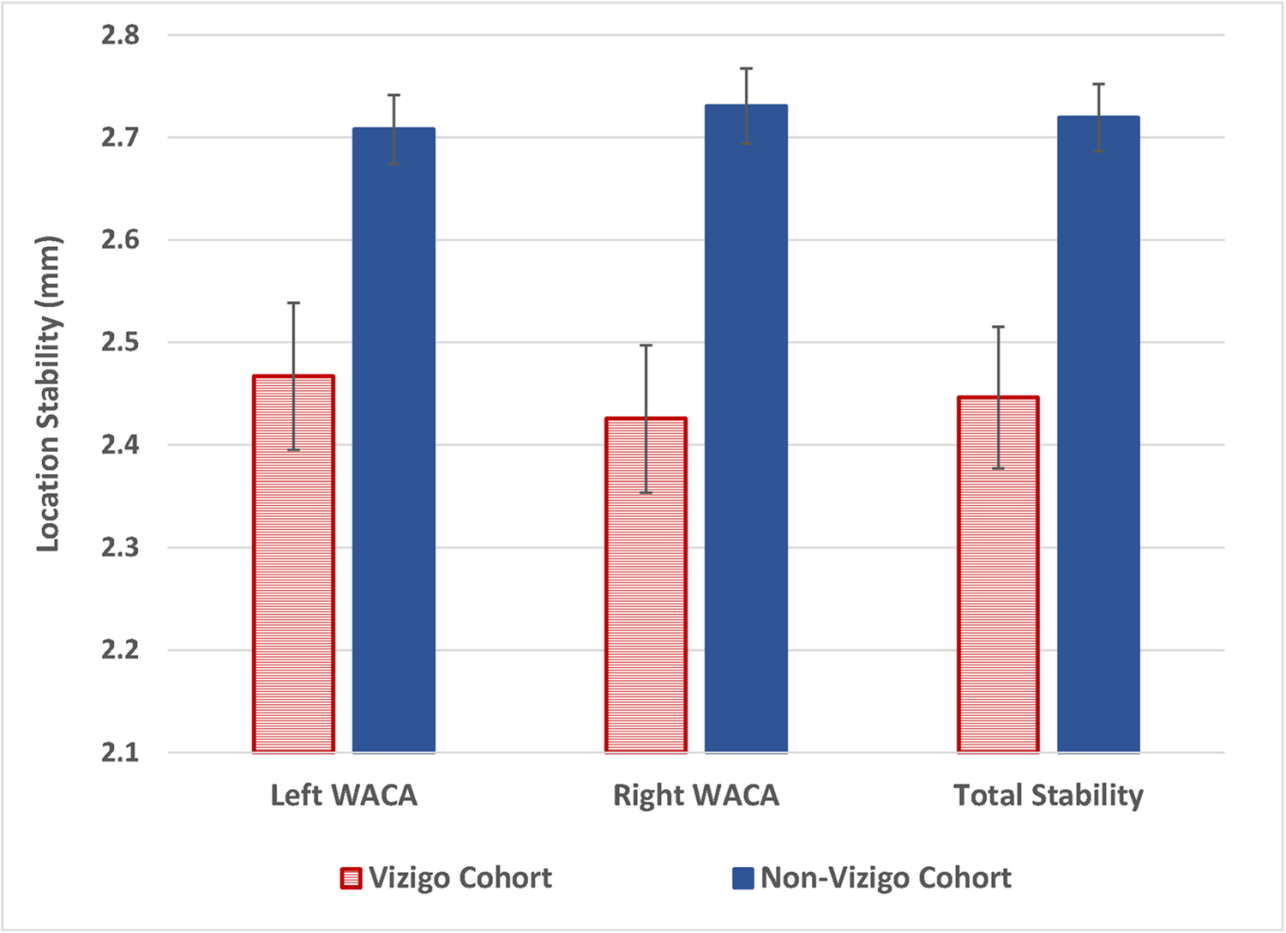
Weighted mean values of catheter location stability (*N* = 256)

In both cohorts, fluoroscopy time was extremely low, with weighted averages of only 0.1 ± 0.6 s in the Vizigo cohort and 1.5 ± 10.5 s in the non-Vizigo cohort (*p* = 0.0740), but significantly more procedures were performed without fluoroscopy in the Vizigo cohort than in the non-Vizigo cohort (99.1% vs. 91.8%, *p* = 0.0030) (Table 2). RF time was also significantly lower in the Vizigo cohort (22.3 ± 8.3 vs. 26.5 ± 10.1 min, *p* < 0.0001), while other procedural efficiency measures were comparable across cohorts. Catheter stability was significantly improved with Vizigo (2.45 mm vs. 2.72 mm overall, *p* = 0.0005), and the improvement was consistent across the left and right PV encirclements (*p* = 0.0027 and *p* = 0.0002) (Fig. 2)

**Table 2:** Weighted procedural outcomes

Procedural outcome	With Vizigo (*N* = 142)	Without Vizigo (*N* = 173)	Weighted *P*-values*
Total procedure time (minutes)	80.7 ± 29.7	79.8 ± 33.9	0.8136
RF time (minutes)	22.3 ± 8.3	26.5 ± 10.1	< 0.0001
PV RF time (minutes)	17.9 ± 6.0	19.0 ± 6.6	0.1382
No fluoroscopy used	99.1%	91.8%	0.0030
Radiation dose (mGy)	0.04 ± 0.29	0.47 ± 1.83	0.28
Total fluids, IV + catheter (mL)	862.4 ± 316.0	892.3 ± 316.2	0.4085
Fluids via catheter (mL)	514.4 ± 217.9	537.8 ± 195.3	0.3154
First pass isolation—left PV encirclement	82.3%	85.8%	0.4091
First pass isolation—right PV encirclement	66.2%	64.3%	0.7256

All observations were weighted by stabilized inverse propensity of treatment weights. Values are reported as mean ± standard deviation for continuous variables and percentages for categorical variables. *P*-values are from chi-square tests for categorical variables and *t*-tests for continuous variables, using the Satterthwaite approximation when variances were unequal across cohorts at a significance level of 0.05

### Complications

Complications were few across cohorts, with no death, atrioesophageal fistula, or stroke. The Vizigo cohort had complications reported in 6 patients, including one arteriovenous fistula, one cardiac tamponade/pericardial effusion, two hematomas (one with bleeding), one pseudoaneurysm, and one case of pericarditis. The non-Vizigo cohort had 2 patients with reported complications, including one cardiac tamponade requiring pericardiocentesis and one pseudoaneurysm requiring femoral artery repair.

### Clinical success

**Fig. 3:**
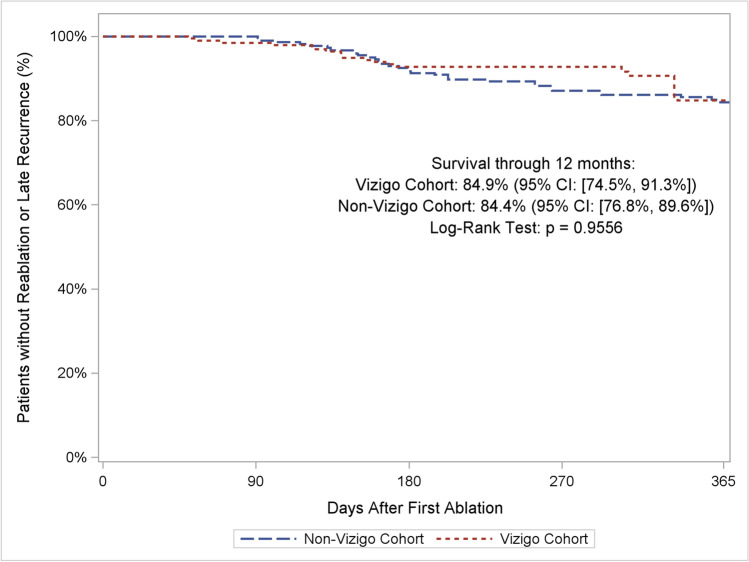
Weighted single-procedure freedom from atrial arrhythmia recurrence

Utilization of the Vizigo sheath did not significantly affect clinical effectiveness outcomes. Weighted Kaplan-Meier estimates of clinical success at 12 months were 84.9% for the Vizigo cohort and 84.4% for non-Vizigo cohort (log-rank *p* = 0.9556) (Fig. 3). Weighted estimates of reablation rates were 3.9% for the Vizigo cohort and 6.2% for the non-Vizigo cohort (chi-square *p* = 0.3601). The impacts of additional patient and procedural predictors on the risk of recurrence are reported in Online Resource 1.

## Discussion

While catheter ablation workflows have traditionally utilized fluoroscopy to monitor catheter location, concerns regarding the acute and chronic consequences of radiation exposure have motivated the transformation of ablation workflows, largely enabled by the availability of 3D EAM systems and real-time intracardiac echocardiography, to minimize fluoroscopy exposure. Furthermore, fluoroscopy provides limited information, as it does not allow visualization of the tissue-catheter interface and is not a real-time continuous imaging modality. Some of the major challenges to the low/zero-fluoroscopy approach are ensuring patient safety, including during transseptal puncture [3], and understanding the relationship between the steerable sheath and ablation catheter. In this study, the Vizigo sheath was not used for the transseptal puncture only because the operators had an established workflow of using the SL2 for that purpose. Incorporating the Vizigo sheath into the workflow as described above was thought to provide the most seamless transition. This study showed that the incorporation of a 3D EAM-visualizable steerable sheath into a minimal fluoroscopy AF ablation workflow can facilitate the transition from low fluoroscopy to zero fluoroscopy, presumably by allowing operators to verify the relative location of the catheter to the sheath without compromising safety or clinical success. Additionally, for operators currently using a conventional fluoroscopy ablation workflow with a contact force sensing catheter, the visualizable sheath may support substantial fluoroscopy reduction.

To date, several observational and randomized studies have demonstrated that well-constructed workflows accompanied by appropriate operator training can facilitate successful transition to low-to-zero fluoroscopy catheter ablation procedures [1, 11, 19–23]. Our results showed that the 3D EAM-visualizable sheath can be incorporated into an existing low/zero fluoroscopy workflow seamlessly, while further reducing the RF ablation time and improving catheter stability. Our results did not show a difference between cohorts in total procedure time, likely due to the already low baseline value which our center has optimized over the past few years combined with the variability of the non-ablation portion of the procedure time. A learning curve in manipulating a steerable sheath may also contribute to procedure time outside of ablation time, as the operators were not using a long sheath for the ablation catheter prior to incorporating the Vizigo sheath into their workflow. In contrast, another recent study found that AF ablation without fluoroscopy was associated with reduced total procedure time, suggesting that less reliance on fluoroscopy for verification of catheter placement in addition to 3D EAM mapping may indeed optimize procedural efficiency [24]. It would be interesting for future studies, especially in academic training centers with less experienced operators/trainees, to understand if procedure time may be improved with the incorporation of the 3D EAM-visualizable sheath.

One of the main incentives for operators to adopt a low fluoroscopy workflow is the ability to reduce their time spent wearing a lead apron during procedures, which has been known to cause long-term orthopedic injuries. In one minimal-to-zero fluoroscopy workflow approach, the majority of operators and other electrophysiology staff were able to remove their lead aprons prior to the first ablation. Specifically, in a zero-fluoroscopy procedure, not only is the operator able to avoid wearing the lead apron completely, but patients and lab staff also benefit from the absence of radiation exposure [11]. Our results suggest that the addition of the new 3D EAM-visualizable sheath can facilitate the transition to zero fluoroscopy in a safe manner, allowing elimination of a lead apron during the procedure.

In addition to an increase in zero-fluoroscopy procedures and shorter RF times, another interesting outcome related to Vizigo usage in our study was the significant improvement in catheter location stability, which is likely responsible for the significant reduction in RF time observed. Though we did not see evidence that this translated to a significant improvement in longer-term clinical outcomes, we did observe a non-significant level of reduction in reablations in the Vizigo cohort. The potential connection may warrant further study based on findings that CF stability is a key factor in improving clinical outcomes [25]. Improved catheter stability may also contribute to improving procedural efficiencies, as less catheter repositioning may be required.

### Limitations

The non-randomized design is the primary limitation of the current study. While real-world observational studies provide valuable information, the inherent quasi-experimental design can be susceptible to confounding. For example, the current study had an imbalance of Vizigo cases across operators at the clinical site due to an unanticipated difference in timing of adopting the new sheath. This confounding necessitated the use of stabilized IPTW for proper statistical inference. This strategy has been proven to result in appropriate statistical estimates while maintaining the sample size of the original data [16], but it is possible with the observational design that additional unknown and unmeasured confounders could remain.

This study was based on data from a single site, which had previously adopted a low-fluoroscopy workflow several years prior to the integration of the Vizigo sheath. Consequently, fluoroscopy usage was seen to be minimal even in the non-Vizigo cohort, suggesting that perhaps improvements in procedural efficiencies and outcomes may be more pronounced at sites using more traditional fluoroscopy workflows. Future multi-site research is needed to confirm generalizability of our observed results.

Finally, this study was not aimed to compare the designs of Vizigo and other sheaths. Further evaluation comparing sheath length and deflectability would help to better elucidate differences among technologies. As such evaluation is scarce, our study helps clarify the visual benefits of Vizigo.

## Conclusions

A low-fluoroscopy ablation workflow incorporating a 3D EAM-visualizable Vizigo sheath was safe and effective in a PAF population. Procedural efficiency was improved, with decreased RF application time and improved catheter stability, while reducing potential health risks associated with radiation exposure for patients and procedure staff.

## Supplementary Information

Below is the link to the electronic supplementary material.

Supplementary file1 (PDF 121 KB)
