# Correction to: Steerable sheath visualizable under 3D electroanatomical mapping facilitates paroxysmal atrial fibrillation ablation with minimal fluoroscopy

**DOI:** 10.1007/s10840-022-01367-x

**Published:** 2022-09-06

**Authors:** Anil Rajendra, Tina D. Hunter, Gustavo X. Morales, Paul Zei, Lee Ming Boo, Allyson Varley, Jose Osorio

**Affiliations:** 1 Alabama Cardiovascular Group, Suite 720, Birmingham, AL 3686 USA; 2 CTI Clinical Trial Services, Covington, KY USA; 3 Brigham and Women’s Hospital, Boston, MA USA; 4 Biosense Webster, Inc, Irvine, CA USA; 5 Heart Rhythm Clinical and Research Solutions, Birmingham, AL USA

**Correction to: Journal of Interventional Cardiac Electrophysiology**

10.1007/s10840-022-01332-8

The article “Steerable sheath visualizable under 3D electroanatomical mapping facilitates paroxysmal atrial fibrillation ablation with minimal fluoroscopy”, written by Anil Rajendra, Tina D. Hunter, Gustavo X. Morales, Paul Zei, Lee Ming Boo, Allyson Varley and Jose Osorio, was originally published electronically on the publisher’s internet portal on 10 August 2022 without open access. With the author(s)’ decision to opt for Open Choice the copyright of the article changed on 21 August 2022 to © The Author(s) 2022 and the article is forthwith distributed under a Creative Commons Attribution 4.0 International License, which permits use, sharing, adaptation, distribution and reproduction in any medium or format, as long as you give appropriate credit to the original author(s) and the source, provide a link to the Creative Commons licence, and indicate if changes were made. The images or other third party material in this article are included in the article’s Creative Commons licence, unless indicated otherwise in a credit line to the material. If material is not included in the article’s Creative Commons licence and your intended use is not permitted by statutory regulation or exceeds the permitted use, you will need to obtain permission directly from the copyright holder. To view a copy of this licence, visit http://creativecommons.org/licenses/by/4.0.

The original article has been corrected.

