# Supplementary material for: Steerable sheath visualizable under 3D electroanatomical mapping facilitates paroxysmal atrial fibrillation ablation with minimal fluoroscopy
Source: J Interv Card Electrophysiol. 2022 Aug 10;66(2):381–8. doi: 10.1007/s10840-022-01332-8 (PMC9977702; doi:10.1007/s10840-022-01332-8)
Supplement: Supplementary file 1 — Supplementary file1 (PDF 121 KB) [file 10840_2022_1332_MOESM1_ESM.pdf]

Supplemental Table. Weighted Predictors of Atrial Arrhythmia Recurrence

Predictor Variable	N	Categorical Predictor Statistics		Continuous Predictor Statistics	
		Weighted Relative Risk of Recurrence	Kaplan-Meier Log-Rank P-Value	Hazard Ratio for Recurrence	Cox Regression Wald Chi-Square P-Value
Patient Characteristics					
Vizigo cohort	315	1.03	0.9556		
Age at procedure	315			1.02	0.2396
Female	315	0.81	0.5019		
Congestive heart failure	315	1.42	0.5197		
Hypertension	315	1.44	0.2593		
Diabetes mellitus	315	0.50	0.1413		
Prior stroke or transient ischemic attack	315	2.78	0.0003		
Vascular disease	315	0.68	0.4097		
CHA_2DS_2-VASc score	315			1.13	0.2338
Left atrial diameter	229			1.25	0.4401
Left ventricular ejection fraction	236			0.99	0.8208
Ablation procedure detail					
Lesion set included any substrate modification	310	4.04	<0.0001		
Substrate modification at posterior wall	315	3.16	0.0063		
Substrate modification at mitral isthmus	315	1.26	0.7719		
Acute ablation outcomes					
First pass isolation of all pulmonary veins	304	0.52	0.0595		
First pass isolation of left pulmonary veins	307	0.45	0.0548		
First pass isolation of right pulmonary veins	306	0.75	0.3881		
Return of conduction with drug challenge	315	0.96	0.9785		

All observations were weighted by stabilized inverse propensity of treatment weights.
